# Comparative Analysis of Acid Sphingomyelinase Distribution in the CNS of Rats and Mice Following Intracerebroventricular Delivery

**DOI:** 10.1371/journal.pone.0016313

**Published:** 2011-01-25

**Authors:** Christopher M. Treleaven, Thomas Tamsett, Jonathan A. Fidler, Tatyana V. Taksir, Seng H. Cheng, Lamya S. Shihabuddin, James C. Dodge

**Affiliations:** Genzyme Corporation, Framingham, Massachusetts, United States of America; Brigham and Women's Hospital, Harvard Medical School, United States of America

## Abstract

Niemann-Pick A (NPA) disease is a lysosomal storage disorder (LSD) caused by a deficiency in acid sphingomyelinase (ASM) activity. Previously, we reported that biochemical and functional abnormalities observed in ASM knockout (ASMKO) mice could be partially alleviated by intracerebroventricular (ICV) infusion of hASM. We now show that this route of delivery also results in widespread enzyme distribution throughout the rat brain and spinal cord. However, enzyme diffusion into CNS parenchyma did not occur in a linear dose-dependent fashion. Moreover, although the levels of hASM detected in the rat CNS were determined to be within the range shown to be therapeutic in ASMKO mice, the absolute amounts represented less than 1% of the total dose administered. Finally, our results also showed that similar levels of enzyme distribution are achieved across rodent species when the dose is normalized to CNS weight as opposed to whole body weight. Collectively, these data suggest that the efficacy observed following ICV delivery of hASM in ASMKO mice could be scaled to CNS of the rat.

## Introduction

Although periodic, intravenous infusions of recombinant lysosomal enzymes has been shown to be effective at resolving the visceral disease associated with a number of lysosomal storage disorders (LSDs) including Gaucher, Fabry, MPS I, MPS II, MPS VI and Pompe disease [1], [2], [3], [4], [5], [6], the blood brain barrier (BBB) has precluded this therapeutic approach from being used to treat LSDs that display CNS neuropathology such as Niemann Pick Type A (NPA) disease. NPA is caused by a deficiency of the lysosomal enzyme acid sphingomyelinase (ASM) and resultant tissue accumulation of non-degraded substrates. Patients with NPA present with hepatosplenomegaly and a progressive neurodegenerative course that typically leads to death by 2 to 3 years of age [7], [8].

With respect to NPA disease, several strategies to overcome the challenge of treating the neuropathic disease have been proposed. These include intracerebral injection of gene, cell and protein based therapies all of which have shown promise when tested in the acid sphingomyelinase knockout (ASMKO) mouse model of NPA. By delivering these therapeutics directly into the brain and thereby bypassing the BBB, the burden of lysosomal storage of sphingomyelin in the CNS was significantly reduced and in many instances resulted in concomitant improvements in motor function [9], [10], [11], [12], [13], [14], [15]. However, except for instances where distal regions were targeted with viral vectors (e.g., AAV vectors) capable axonal transport, correction of lysosomal storage pathology was typically localized to areas that were proximal to the injection site. Given that NPA is a global neurometabolic disease, the development of therapeutic delivery strategies that result in widespread correction of disease pathology throughout the CNS is clearly desirable. One potential strategy for achieving broad lysosomal enzyme distribution throughout the CNS is to exploit the natural flow of cerebrospinal fluid (CSF) through either intrathecal or intracerebroventricular (ICV) enzyme administration. Indeed, we recently reported that ICV infusion of recombinant human acid sphingomyelinase (hASM) into ASMKO mice led to a successful reduction in the pathological accumulation of both sphingomyelin and cholesterol throughout the CNS [16]. In light of these encouraging results we sought to confirm that these observations were translatable to species with a larger and more complex nervous system. Therefore, in our current experiments we determined the following: (1) the extent to which regions proximal and distal to the infusion site can be targeted in the rat CNS; (2) the relationship between the level of hASM detected in the rat CNS and the dose infused; (3) the percentage of the total dose detected in the rat CNS parenchyma; and (4) the relative distribution of hASM in the rat CNS (when compared to the mouse CNS) when the scale up dose is normalized to body weight vs. CNS weight. Our findings indicate that ICV infusion of hASM is scalable from the mouse to the rat CNS and that further studies to extend these observations to yet larger animal models, such as non-human primates, is warranted.

## Results

### ICV infusion of hASM into rats results in widespread distribution of the enzyme throughout the CNS

**Figure 1:**
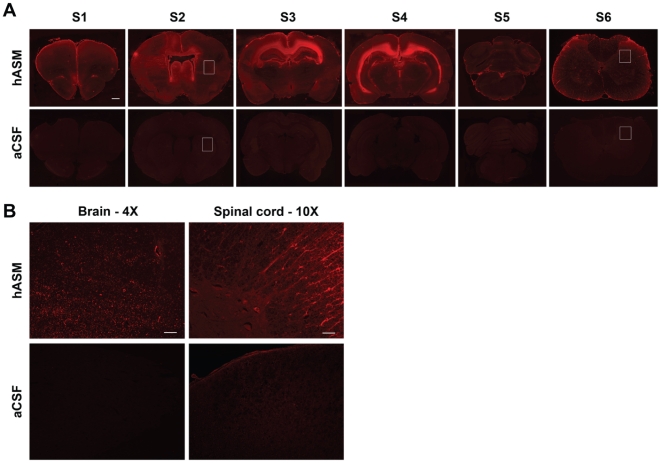
(A) Distribution of hASM in the rat CNS following a 6 h ICV infusion of hASM or aCSF (S1 = most rostral brain section and S5 = most caudal brain section, S6 = cervical spinal cord). Positive hASM staining was observed throughout the brain and spinal cord of rats administered with the enzyme. Staining intensity was maximal in regions proximal to ventricular compartments and the sub-arachnoid space. (B) Higher magnification (4× for brain; 20× for spinal cord) of hASM staining in regions between ventricular compartments and the tissue proximal to the sub-arachnoid space (area enclosed within the white box of sections S2 and S6). Shown are images from the brain and cervical spinal cord.

**Figure 2:**
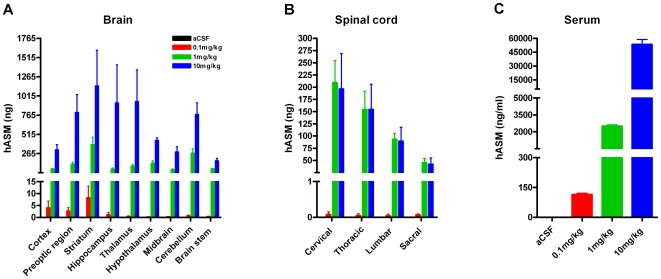
hASM levels in (A) rat brain, (B) spinal cord and (C) serum following a 6 h ICV infusion of hASM at different doses (0.1, 1.0 and 10 mg/kg) or aCSF. Significant (p<.01) levels of hASM were detected in all regions of the brain (i.e., cortex, preoptic region, striatum, hippocampus, thalamus, hypothalamus, midbrain, cerebellum and brainstem) in rats infused with 1 and 10 mg/kg of the enzyme. Rats infused with the 0.1 mg/kg dose displayed significant hASM levels in the cortex, preoptic region, striatum and hippocampus. No significant differences in hASM levels between regions were observed regardless of the dose tested. In each region of the spinal cord, significant (p<.01) levels of hASM were also detected in rats infused at either 1 or 10 mg/kg vs. rats infused with aCSF (Figure 2b). Detected hASM levels in rats infused with 0.1 mg/kg were not significantly above background. Significant (p<.01) levels of hASM were detected in serum for all hASM infused rats regardless of the dose of enzyme used.

Female Sprague Dawley rats (n = 3/group) were infused with either hASM (10 mg/kg) or artificial cerebrospinal fluid (aCSF) and then killed immediately for immunohistochemical (IHC) analysis to determine enzyme distribution within the CNS. In enzyme-treated rats, positive hASM immunostaining was observed throughout the rostral-caudal extent of the brain. Areas that were either adjacent to the site of administration (ventricles) or to the subarachnoid space surrounding the cortex displayed the strongest signals (Figure 1a, S1–S6). Examination of the regions between the ventricles and areas proximal to the subarachnoid space at a higher magnification (4× for brain) showed these regions also stained positively for hASM, albeit at a lower intensity (Figure 1b). The pattern of hASM staining in the spinal cord displayed a similar gradient of signal intensity. Positive immunostaining was localized throughout each division (cervical, thoracic, lumbar and sacral) of the spinal cord; however, regions immediately adjacent to the central canal of the spinal cord or to the subarachnoid space surrounding the spinal cord displayed the highest intensity of staining (Figure 1a, S6). To determine if hASM could be detected in CNS tissue in a dose dependent manner, cohorts of rats (n = 6/group) were infused with either hASM (at a dose of 0.1, 1.0 or 10 mg/kg) or with aCSF and were then killed immediately for ELISA analysis. Levels of hASM in all regions of the brain (i.e., cortex, preoptic region, striatum, hippocampus, thalamus, hypothalamus, midbrain, cerebellum and brainstem) of rats infused with 1 and 10 mg/kg of the enzyme were significantly higher than noted in the control aCSF-treated animals (Figure 2a). Rats infused with the lowest dose of hASM (0.1 mg/kg) showed higher hASM levels (above background) only in the cortex, preoptic region, striatum and hippocampus. While the levels of hASM in the different regions of the brain were not significantly different from each other, rats administered 10 mg/kg hASM showed a trend towards higher levels in areas that were adjacent to the lateral ventricles (e.g., striatum, hippocampus and thalamus). Interestingly, although a dose response was observed, this relationship was not linear. For example, rats treated with 10 mg/kg of hASM only showed a 3 to 6 fold increase in hASM levels in most brain regions when compared to rats that were infused with 1 mg/kg of enzyme (Figure 2a).

All divisions of the spinal cord of rats infused with 1 and 10 mg/kg hASM showed significantly higher (p<0.01) levels of the enzyme when compared to rats infused with aCSF (Figure 2b). Animals administered the lowest dose of 0.1 mg/kg hASM did not exhibit measurable amounts of enzyme above background levels. The highest levels of hASM were detected in the cervical region and the lowest were in the sacral division (Figure 2b). The thoracic and lumbar regions showed intermediate levels. Similar to what was observed in the brain, a non-linear dose dependent relationship was found in the spinal cord. In fact, hASM levels detected in the spinal cord of rats infused with 1 and 10 mg/kg were equivalent despite the 10-fold difference in dose (Figure 2b). These data suggest that a threshold amount of enzyme may need to be administered before broad enzyme distribution can be attained in the CNS. In the spinal cord, ICV infusion of 1 mg/kg may be sufficient to maximize the delivery to this region.

### Significant levels of hASM are detected in serum following ICV infusion into rats

Given that CSF circulates back into venous circulation we also analyzed serum for levels of hASM immediately following the completion of the infusion procedure. In female rats hASM has a ½ life of approximately 70 minutes (unpublished observations). Significant (p<0.01) levels of hASM were detected in serum in all hASM-infused rats regardless of the dose used (Figure 2c). Interestingly, in contrast to what was observed in the CNS, a linear dose-dependent relationship was observed in hASM-treated rats. A corresponding 10-fold difference (or greater) in hASM levels was observed between rats administered 0.1 and 1.0 mg/kg hASM and between animals treated with 1.0 and 10 mg/kg of the enzyme.

### The absolute level of hASM detected in CNS tissue following ICV infusion represents a small percentage of the total infused dose

**Figure 3:**
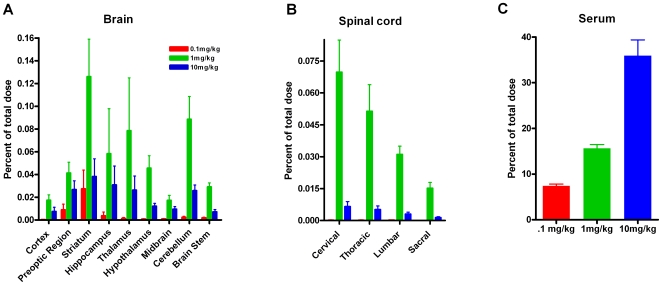
Percentage of the total administered dose of hASM detected in the (A) brain, (B) spinal cord and (C) serum of rats following 6 h ICV infusion. Animals were infused with 0.1, 1.0 or 10 mg/kg of hASM or aCSF. For both the brain and spinal cord, less than 0.2% of the total dose could be detected in each region. Cumulatively, the level of hASM detected in the brain and spinal cord was less than 1% of the total dose administered. No significant differences were observed between regions or different dose groups. Significant (p<.01) levels of hASM were detected in serum for each dose.

To determine how much of the total infused dose diffused into the parenchyma of the CNS, the percent of the total hASM delivered into different brain regions and spinal cord was calculated. Interestingly, in both the brain and spinal cord only a very small fraction (less than 0.2% of the total dose) was detected in each of the regions measured (Figures 3a, 3b). Cumulatively, the level of hASM delivered into the brain and spinal cord was less than 1% of the total dose administered. Comparison of data from rats treated with 1 and 10 mg/kg doses indicated that delivering a higher dose resulted in a smaller percentage of the enzyme being delivered into the CNS (Figure 3a and 3b). This observation correlates with the findings of a non-linear dose response and suggests that at the 10 mg/kg dose, we may have saturated the processes responsible for the uptake of the enzyme. However, it is unlikely that higher levels of hASM would have been detected in CNS tissue if the interval between the termination of the infusion procedure and tissue collection had been increased. This notion is based on prior observations (unpublished) which have showed that maximal levels of hASM are detected within visceral tissue (e.g., liver, kidney, spleen and lung) almost immediately following systemic administration. A non-linear dose response was also reflected by the amounts of enzyme that were detected in the serum (Figure 3c). Interestingly, as the dose of hASM increased 10 - fold the percentage of enzyme detected in serum approximately doubled for subsequent dose (0.1 mg/kg = 7.4%, 1.0 mg/kg = 15.6% and 10 mg/kg = 35.9%; Figure 3c).

### Similar levels of hASM are detected in the CNS of rats and mice when the dose is normalized to CNS weight rather than body weight

**Figure 4:**
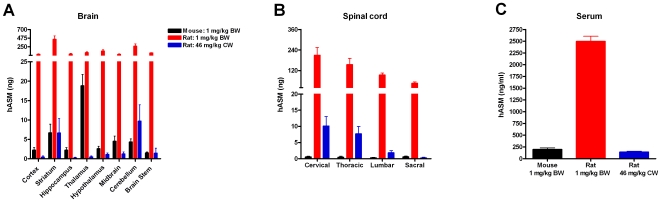
Comparison of hASM levels in the (A) brain, (B) spinal cord and (C) serum of rats and mice infused at equivalent doses normalized to body weight (BW) and CNS weight (CW). Mouse BW dose of 1 mg/kg is approximately equal to 46 mg/kg/CW when converted. ICV hASM infusion in mice (at 1 mg/kg/BW or 46 mg/kg/CW) resulted in significant (p<.01) enzyme distribution throughout all of the brain regions examined (i.e., cortex, striatum, hippocampus, thalamus, hypothalamus, midbrain, cerebellum and brainstem) vs. aCSF infused mice. hASM levels in the mouse spinal cord were not significantly above background. Rats infused at equivalent dose based on BW (1.0 mg/kg) displayed levels of hASM that were significantly (p<.01) several fold higher for both the brain and spinal cord. hASM levels in rats infused at an equivalent CW dose (46 mg/kg) were comparable to levels detected in the mouse CNS. Serum hASM levels were significantly (p<.01) several fold higher in rats vs. mice infused at comparable BW dose, whereas rats infused at an equivalent CW dose displayed serum hASM levels that were not significantly different from levels detected in mice.

Previously, we reported that ICV infusion of 1 mg/kg hASM was the lowest effective dose to reduce lysosomal accumulation of sphingomylein (the primary substrate of hASM) in a mouse model of Niemann Pick A disease [16]. In the current study, we measured hASM levels attained within specific regions of the mouse CNS following infusion at this same dose to determine the tissue levels needed for the observed efficacy (n = 8). It is currently unclear, however, if species scale up doses should be normalized to body weight (BW) which is 10–15 fold greater in rats (assuming rat BW = 0.3 kg) or CNS weight (CW) which is approximately 5 fold greater in rats (assuming rat CW = 0.028 kg). Therefore, to determine which dose scaling metric should be employed for ICV enzyme infusion, the results of the current mouse experiment were compared with the findings observed in rats infused with 1 mg enzyme/kg of BW (i.e., 0.3 mg total dose) and rats (n = 6) that were administered a dose of 46 enzyme mg/kg of CW (i.e., 0.128 mg total dose). In ASMKO mice 46 mg/kg of CW is the lowest effective dose when the dose is normalized to CW. ICV infusion of hASM into mice (at 1 mg/kg of BW or 46 mg/kg when normalized to CW) resulted in significant (p<.01) enzyme distribution throughout all brain regions examined (i.e., cortex, striatum, hippocampus, thalamus, hypothalamus, midbrain, cerebellum and brainstem; Figure 4a). In contrast to the brain, the levels of hASM in the mouse spinal cord were not significantly above background (noted in matched tissues from aCSF-infused mice) in the majority of animals analyzed (Figure 4b). Rats infused with an equivalent dose based on BW (1.0 mg/kg) displayed levels of hASM that were significantly (p<0.01) higher in both the brain and spinal cord than what was noted in mice treated with hASM (Figure 4a, 4b). Interestingly, when rats were administered an equivalent dose based on CW (46 mg/kg) the levels of hASM attained in the majority of CNS regions analyzed were fairly comparable between species (Figure 4a, 4b). The only notable differences were in the thalamus and in the spinal cord. Similar to the observations in the CNS, serum hASM levels were also significantly (p<.01) higher in rats than in mice when the dose administered had been normalized based on their BW. Rats that were infused at a dose that had been normalized based on their CW displayed serum hASM levels that were not significantly different from the levels detected in mice (Figure 4c).

## Discussion

The blood brain barrier (BBB) has proven to be a formidable obstacle in the development of therapeutic treatments for several neurodegenerative disease including neuropathic lysosomal storage diseases (LSDs) such as Niemann Pick Type A (NPA) disease. Circumventing the BBB through intracerebroventricular (ICV) drug administration has been reported to be a viable approach for addressing several CNS disease states including brain infection, brain tumors, and intracerebral hemorrhage [17], [18], [19], [20], [21]. Indeed, recently we had reported that ICV infusion of recombinant human acid sphingomyelinase (hASM) in a mouse model of NPA disease (i.e., ASMKO mice) led to widespread enzyme delivery throughout the CNS in a manner that significantly reduced pathological lysosomal substrate accumulation in the brain and spinal cord [16]. Therefore, in our current experiments we further explored the translatability of this therapeutic delivery approach by comparing the distribution of hASM in the rat and mouse CNS following ICV infusion.

Translation of therapeutic observations made in mouse models of CNS disease to a non-human species with a larger and more complex nervous system is in most instances a prerequisite to testing an experimental therapy in man. Testing CNS drugs in rats has proven to be a useful intermediate step to assess a therapeutic agent's permeation and distribution within the CNS prior to completing similar and more expensive experiments in non-human primates. Brain pharmacokinetic studies performed in rats have the potential to be especially useful when such experiments are linked to neurochemical, physiological, behavioural or neuroimaging readouts that support target activation; however, the first step is to demonstrate adequate distribution of the therapeutic agent (i.e., at a concentration shown to be efficacious in mouse models of the disease) within target regions of the CNS. In the case of neuropathic LSDs such as NPA, the target region is the entire CNS as NPA is a global neurometabolic disease.

Immunohistochemical (IHC) examination of the rat CNS following ICV infusion (at a concentration of 10 mg/kg) showed the enzyme was widely distributed throughout the rostral-caudal extent of both the brain and spinal cord. Interestingly, the intensity of hASM staining was strongest in regions that were proximal to the ventricle and the sub-arachnoid space surrounding the brain. This pattern of staining was not readily apparent in all CNS regions analyzed in our prior ASMKO mouse studies [16]. In agreement with the IHC findings, ELISA measurements revealed significant levels of hASM throughout discrete regions of the brain (i.e., cortex, preoptic region, striatum, hippocampus, thalamus, hypothalamus, midbrain, cerebellum and brainstem) and each division of the spinal cord. Although there were no significant regional variations at the whole tissue level, there was a trend for detecting higher levels of hASM in regions that were proximal to the lateral ventricles (e.g., hippocampus). Similar findings were made in the spinal cord, as a gradient of hASM was observed with the highest levels being in the cervical and the lowest in sacral divisions.

The effect of administering increasing doses of hASM on its distribution in the CNS was also examined. Although ICV infusion of hASM at lower doses (i.e., 0.1 and 1.0 mg/kg) resulted in measureable amounts of hASM within the CNS (depending on the dose and brain region analyzed), the level of hASM detected was not directly proportional to the dose infused. For example, when hASM levels were compared in rats dosed at 10 mg/kg vs. 1 mg/kg only a 3–6 fold increase in detected hASM levels was found in most brain regions analyzed despite the fact a 10-fold higher dose was employed. Interestingly, rats treated with 1.0 and 10 mg/kg hASM displayed equivalent levels of hASM throughout the spinal cord. These results suggest that the amount of hASM that diffuses into CNS parenchyma (especially to the spinal cord) or that undergoes cellular uptake following ICV infusion is limited and is not linearly dependent upon the concentration of the enzyme delivered to the CSF compartment. Several additional findings from our current studies support this view. Specifically, we found that less than 1.0% of the total dose delivered to the lateral ventricle was detected in CNS tissue regardless of the dose infused. In addition, a higher percentage of the total dose administered was detected in CNS tissues of rats treated with lower vs. higher doses (e.g., 1.0 vs. 10 mg/kg). Moreover, a significant dose-dependent increase in serum hASM levels was observed following ICV infusion. The latter finding was expected given that CSF flows out of the ventricular system into the subarachnoid space and into the venous circulation in part through the arachnoid villi in the venous sinuses [22], [23] Although we found significant levels of hASM in serum following ICV infusion, a significant percentage of the total dose still remained unaccounted for. It is likely that a significant percentage of the infused enzyme was internalized by the visceral tissues and/or delivered to the lymphatic system. This supposition is supported by our earlier work demonstrating that ICV infusion of hASM into ASMKO mice led to a significant reduction in lysosomal accumulation of sphingomyelin in several visceral organs including the spleen, liver and lungs [16]. Although we have not assessed this directly, it is expected that a significant amount of hASM would also be found in the lymphatic system as it serves as a major drainage route for CSF leaving the CNS [22], [23], [24].

A number of cellular and diffusion barriers (e.g., size and charge of hASM, enzymatic inactivation, and sequestration of the enzyme by binding proteins) may have been responsible for limiting the penetration and diffusion of hASM into surrounding CNS parenchyma following ICV infusion. Although neighboring ependyma cells are linked by gap rather than tight junctions to facilitate the outflow of endogenous polypeptides (e.g., leptin and insulin) from the CSF to the interstitial fluid [25], [26], the relatively large size of hASM (72 kDa) may have restricted its penetration into CNS tissue. For example, it has been reported that smaller, hydrophilic peptides (e.g., leptin, 16 kDa) can be widely distributed throughout the CNS upon ICV delivery whereas larger polypeptides (e.g., FGF-2, 22 kDa; GDNF, 24 kDa) which also have an affinity for components (e.g., heparan sulfate) of the extracellular matrix have a more limited distribution [26], [27]. Alternatively, detection of low levels of hASM in CNS parenchyma may be due to accelerated removal of hASM through efflux pumps (e.g., p-glycogprotein multidrug-resistance pump); as it has been suggested that ependymal cells possess both the structural and enzymatic machinery that is necessary for scavenging a wide variety of substances in the CSF to form a metabolic barrier at the brain-CSF interface [25]. Enzymatic inactivation of the infused hASM by the choroid plexus (CP) may have also been responsible given that the CP expresses a number of different peptidases [26]. Finally, it is also possible that enzyme diffusion may not be limiting and that only a small percentage of the total dose is detected in CNS tissue because the cellular uptake of hASM, which is mannose 6-phosphate receptor (M6PR)-mediated [28], [29], becomes saturated after a given enzyme concentration is achieved (i.e., when maximal M6PR occupation occurs or after enzyme internalization becomes saturated). Clearly additional experiments are necessary to determine M6PR distribution within CNS tissue, which cell types express M6PR receptors, the dose at which M6PRs reach saturation and the ½ life of hASM in CNS parenchyma in order to better understand the potential of ICV enzyme infusion as a therapeutic delivery approach for the treatment of neuropathic LSDs. However, we believe that these questions will best be answered in an animal model (e.g., non human primate) with a CNS composition that is more similar to human.

Nevertheless, despite the fact that only a small percentage of the total ICV administered dose was detected in rat CNS parenchyma, hASM was distributed throughout the CNS and at a level (2–10 ng/region) that is predicted to be within the therapeutic range necessary to be efficacious in the ASMKO mouse CNS [16]. We also demonstrated for the first time that equivalent levels of enzyme are detected in CNS parenchyma and serum when the administered dose was normalized to CNS weight rather than body weight. Prior to our current experiments guidance from the literature indicating which metric should be used for calculating a species scale up dose for ICV enzyme infusion was lacking. This information may prove to be particularly useful in designing experiments that test ICV enzyme infusion in yet larger animal models. This finding is also important from a safety perspective because the physical chemical properties (i.e., osmolarity, pH and the presence of preservatives and diluents) of potential therapeutics can present serious challenges (e.g., initiate seizures, hyperaesthesia and contribute to tachyphlaxis) to whether or not a potential drug therapy can be safely and effectively administered through the ICV delivery route [18].

In conclusion, our results indicate that ICV infusion leads to widespread hASM delivery throughout the CNS in rats at a level shown to be efficacious in ASMKO mice. More experimentation is needed to understand the factors limiting the delivery of the enzyme to the CNS parenchyma following ICV infusion and whether the diffusion/cellular uptake barriers will preclude delivery of clinically relevant levels of hASM to a species with a brain mass and complexity that is more similar to humans (e.g. non- human primate).

## Methods

### Animals

Thirty-six female Sprague Dawley rats (8 to 10 weeks old) and sixteen female C57BL/6 mice (8 to 10 weeks old) were obtained from Charles River Laboratories (Wilmington, MA), maintained under a 12 hr/12 hr light/dark cycle and given water and food ad libitum throughout the study period. All procedures were performed using a protocol (08-1218-03) that had been approved by the Institutional Animal Care and Use Committee at Genzyme.

### Stereotaxic surgery

Mice and rats were anesthetized with 3% isofluorane and placed in a stereotaxic frame for placement of an indwelling guide cannula (Plastics One, Roanoke, VA) to a position that was 1 mm dorsal to the right lateral ventricle (mouse coordinates: A–P: −.4 from bregma, M–L: −1.0 from bregma, D–V: −1.05 from dura, incisor bar: 0.0; rat coordinates: A–P: −1.25 from bregma, M–L: −1.4 from bregma, D–V: −3.2 from dura, incisor bar: 0.0). Guide cannulas were permanently affixed to the skull using anchor screws (CMA Microdialysis Inc., North Chelmsford, MA) and dental acrylic (CMA Microdialysis Inc., North Chelmsford, MA). A dummy cannula (Plastics One, Roanoke, VA) was inserted into the guide cannula to maintain cannula patency prior to insertion of the infusion probe. One hour before and 24 h after surgery, rodents were given ketoprofen (5 mg/kg, administered subcutaneously) for analgesia.

### Intracerebroventricular infusion

On the day of enzyme infusion, the dummy cannula was removed and replaced with an infusion probe (Plastics One, Roanoke, VA) that extended 1 mm ventral to the tip of the guide cannula. Infusion probes were connected to a swivel (Instech Laboratories Inc., Plymouth Meeting, PA) using a tubing that permitted 360 degrees of movement during the infusion procedure. Swivels were connected with tubing to a Hamilton gastight syringe mounted on a Harvard Apparatus infusion pump (Harvard Apparatus, Holliston, Massachusetts) set to deliver enzyme (at variable concentrations depending on the experiment) at a rate approximately equivalent to a quarter of the normal rate of turnover of endogenous CSF (mice: 5 ul/h; rats: 20 ul/h) for up to 6 h. Upon completion of the infusion procedure, infusion probes were then removed and replaced with the dummy cannulas.

### Tissue collection and processing

Animals were killed immediately following the infusion procedure according to a humane protocol approved by the Institutional Animal Care and Use Committee. Following an anesthetic over dose with euthasol, rodents underwent a terminal retro-orbital eye bleed to collect serum for subsequent ELISA of hASM. Animals destined for biochemical analysis were perfused through the heart with phosphate-buffered saline (PBS) to remove all blood. Brain and spinal cord tissue were harvested and snap frozen in liquid nitrogen as reported previously [9], [11]. Prior to being snap frozen, brain tissue was dissected into the following regions: cortex, striatum, preoptic region, hippocampus, hypothalamus, thalamus, midbrain, cerebellum and brainstem. Similarly, the spinal cord was dissected into cervical, thoracic, lumbar and sacral segments prior to being snap frozen. Animals destined for histological analyses were perfused with 4% paraformaldehyde, and the tissues sectioned on a vibratome as reported previously [12].

### Histological and biochemical assays

Brain sections were stained for hASM using a biotinylated anti-hASM monoclonal antibody (Genzyme, Framingham, MA) at a dilution of 1∶200 and visualized as reported [12]. An ELISA specific for hASM (Genzyme, Framingham, MA) was used to quantify the level of the enzyme in serum samples as reported [9].

### Statistics

Levels of hASM in the tissues and serum were analyzed with ANOVAs. Follow-up analyses were conducted with a Bonferroni post-hoc test where appropriate. All values were considered significant if p<0.05.

## References

[1] Amalfitano A, Bengur AR, Morse RP, Majure JM, Case LE (2001). Recombinant human acid alpha-glucosidase enzyme therapy for infantile glycogen storage disease type II: results of a phase I/II clinical trial.. Genet Med.

[2] Barton NW, Furbish FS, Murray GJ, Garfield M, Brady RO (1990). Therapeutic response to intravenous infusions of glucocerebrosidase in a patient with Gaucher disease.. Proc Natl Acad Sci U S A.

[3] Barton NW, Brady RO, Dambrosia JM, Di Bisceglie AM, Doppelt SH (1991). Replacement therapy for inherited enzyme deficiency—macrophage-targeted glucocerebrosidase for Gaucher's disease.. N Engl J Med.

[4] Harmatz P, Whitley CB, Waber L, Pais R, Steiner R (2004). Enzyme replacement therapy in mucopolysaccharidosis VI (Maroteaux-Lamy syndrome).. J Pediatr.

[5] Kakkis ED, Muenzer J, Tiller GE, Waber L, Belmont J (2001). Enzyme-replacement therapy in mucopolysaccharidosis I.. N Engl J Med.

[6] Schiffmann R, Kopp JB, Austin HA, Sabnis S, Moore DF (2001). Enzyme replacement therapy in Fabry disease: a randomized controlled trial.. Jama.

[7] Leventhal AR, Chen W, Tall AR, Tabas I (2001). Acid sphingomyelinase-deficient macrophages have defective cholesterol trafficking and efflux.. J Biol Chem.

[8] Schuchman EH, Desnick RJ, Scriver CR, Beaudet AL, Sly WS, Valle D (2001). Niemann-Pick disease types A and B: acid sphingomyelinase deficiencies.. In The metabolic and molecular basis of inherited Disease. 8th ed.

[9] Dodge JC, Clarke J, Song A, Bu J, Yang W (2005). Gene transfer of human acid sphingomyelinase corrects neuropathology and motor deficits in a mouse model of Niemann-Pick type A disease.. Proc Natl Acad Sci U S A.

[10] Passini MA, Macauley SL, Huff MR, Taksir TV, Bu J (2005). AAV vector-mediated correction of brain pathology in a mouse model of Niemann-Pick A disease.. Mol Ther.

[11] Passini MA, Bu J, Fidler JA, Ziegler RJ, Foley JW (2007). Combination brain and systemic injections of AAV provide maximal functional and survival benefits in the Niemann-Pick mouse.. Proc Natl Acad Sci U S A.

[12] Shihabuddin LS, Numan S, Huff MR, Dodge JC, Clarke J (2004). Intracerebral transplantation of adult mouse neural progenitor cells into the Niemann-Pick-A mouse leads to a marked decrease in lysosomal storage pathology.. J Neurosci.

[13] Yang WW, Dodge JC, Passini MA, Taksir TV, Griffiths D (2007). Intraparenchymal injections of acid sphingomyelinase results in regional correction of lysosomal storage pathology in the Niemann-Pick A mouse.. Exp Neurol.

[14] Jin HK, Carter JE, Huntley GW, Schuchman EH (2002). Intracerebral transplantation of mesenchymal stem cells into acid sphingomyelinase-deficient mice delays the onset of neurological abnormalities and extends their life span.. J Clin Invest.

[15] Jin HK, Schuchman EH (2003). Ex vivo gene therapy using bone marrow-derived cells: combined effects of intracerebral and intravenous transplantation in a mouse model of Niemann-Pick disease.. Mol Ther.

[16] Dodge JC, Clarke J, Treleaven CM, Taksir TV, Griffiths DA (2009). Intracerebroventricular infusion of acid sphingomyelinase corrects CNS manifestations in a mouse model of Niemann-Pick A disease.. Exp Neurol.

[17] Anderson VC, Burchiel KJ (1999). A prospective study of long-term intrathecal morphine in the management of chronic nonmalignant pain.. Neurosurgery.

[18] Cook AM, Mieure KD, Owen RD, Pesaturo AB, Hatton J (2009). Intracerebroventricular administration of drugs.. Pharmacotherapy.

[19] Obbens EA, Leavens ME, Beal JW, Lee YY (1985). Ommaya reservoirs in 387 cancer patients: a 15-year experience.. Neurology.

[20] Luer MS, Hatton J (1993). Vancomycin administration into the cerebrospinal fluid: a review.. Ann Pharmacother.

[21] Haines SJ, Lapointe M (1998). Fibrinolytic agents in the treatment of intraventricular hemorrhage in adults.. Crit Rev Neurosurg.

[22] Kapoor KG, Katz SE, Grzybowski DM, Lubow M (2008). Cerebrospinal fluid outflow: an evolving perspective.. Brain Res Bull.

[23] Tripathi R (1974). Tracing the bulk outflow route of cerebrospinal fluid by transmission and scanning electron microscopy.. Brain Res.

[24] Slusarczyk K, Slusarczyk R, Kiwic G (1996). Lymphatic outflow of the cerebrospinal fluid in rats.. Folia Morphol (Warsz).

[25] Del Bigio MR (1995). The ependyma: a protective barrier between brain and cerebrospinal fluid.. Glia.

[26] Smith DE, Johanson CE, Keep RF (2004). Peptide and peptide analog transport systems at the blood-CSF barrier.. Adv Drug Deliv Rev.

[27] Mufson EJ, Kroin JS, Sendera TJ, Sobreviela T (1999). Distribution and retrograde transport of trophic factors in the central nervous system: functional implications for the treatment of neurodegenerative diseases.. Prog Neurobiol.

[28] Hurwitz R, Ferlinz K, Vielhaber G, Moczall H, Sandhoff K (1994). Processing of human acid sphingomyelinase in normal and I-cell fibroblasts.. J Biol Chem.

[29] Ioannou YA, Bishop DF, Desnick RJ (1992). Overexpression of human alpha-galactosidase A results in its intracellular aggregation, crystallization in lysosomes, and selective secretion.. J Cell Biol.

